# Supplementary material for: Learning curve and surgical outcome of robotic assisted colorectal surgery with ERAS program
Source: Sci Rep. 2022 Nov 29;12:20566. doi: 10.1038/s41598-022-24665-w (PMC9709162; doi:10.1038/s41598-022-24665-w)
Supplement: Supplementary file 2 — Supplementary Information 2. [file 41598_2022_24665_MOESM2_ESM.zip › RAL Raw data-Ñ[▒K/078/Patho_ñw╝╨░O▒Kñσ.pdf]

臺中榮民總醫院 臺中市臺灣大道四段1650號 病理部活體組織切片 臨床醫學實驗及病理診斷中心 檢驗(查)報告

[redacted]

Pathologic diagnosis:
Colon, terminal ileum, and appendix, robotic total colectomy --- a. Submucosal congestion and lymphoid hyperplasia, of terminal ileum and colon. No tumor seen. b. Fibrous obliteration of appendix.

Gross description:
The specimen consists of a segment of colon, 65 cm in length, and 6 cm in proximal circumference, 4 cm in distal cut end circumference, and a segment of terminal ileum, 4 cm in length, and 2.5 cm in cut end circumference. An unremarkable appendix, 5x0.4 cm, is noted. Some erythromatous change over the mucosa is noted and no mass lesion is identified grossly. The appendix, ileocecal valve, and both cut ends are free. Representative part for section: A) proximal cut end B) distal cut end C) ileocecal valve D) appendix E-H) colon mucosa.
#T-59420_2 #M-36100_2 0 1105 000000
#T-59200_2 #M-78250_2 0 1105 000000

已電子簽章

敬告：本單僅供醫師參考使用，不具任何申請效力！！
請病患妥善保管，若不慎遺失需自負個人資料外洩責任！
